# The Dental Protective Association

**Published:** 1890-03

**Authors:** Wm. Jarvie

**Affiliations:** Secretary


### EDITORIAL.

### The Dental Protective Association.

We cheerfully give place to the following statement and resolution, not only to make record of what was done in New York on that occasion, but as an evidence of a growing interest in the work of the Dental Protective Association throughout the country. The evils which called forth this association have existed for a long time, but such was the apathy of the profession in regard to this subject, that it has assumed proportions that are alarming, and that without arrest will become a great burthen. The entire subject of patents, nostrums, and secret remedies, could, with great propriety, and should, receive the attention of such an association, and thereby the profession would derive great benefit.

It would be well if the profession could have, either through the Protective Association, or the American Dental Association, or both jointly, a commission consisting of one or more experts who should examine and decide upon the merits of every new invention, secret process or material, whether patented or not. Such a course would afford immense protection to the profession. There are some, of course, who would prefer to decide for themselves, but the large proportion of the profession would avail themselves of the decision of such a commission and be vastly profited thereby. Such a method would be the means of weeding out all worthless things, and would greatly enhance the value, success and usefulness of the really meritorious.

This method could be further expanded so as to embrace a reasonable compensation to all who should bring forth or invent any thing new and really meritorious. Such a method would be more satisfactory to the inventors than the present, and certainly more beneficial to the profession. For then every one would have some assurance of the value of any new device or process before adopting it in practice. It would also prevent the fruitless waste of money in harassing litigation. We would suggest that every member of the profession who values it, for what it is or for what it may become, or who appreciates what would be for his own interest, should secure membership in the Protective Association, and then have a voice in respect to adopting and carrying out some such scheme as is indicated above.

NEW YORK, January 16th, 1890.

At a mass meeting of over one hundred dentists, gathered from various parts of the United States, held in the city of New York, January 16th, 1890, of which Dr. O. E. Hill, was chairman, it was, on motion, unanimously

*Resolved*, That we thoroughly endorse the Dental Protective Association of the United States, and urge upon every member of the dental profession to join the association and send to Dr. J. N. Crouse, of Chicago, its president, the initiation fee of ten dollars. WM. JARVIE, Secretary.

